# Draft genome sequence of an *Atlantibacter hermannii* clinical strain isolated from human muscle tissue

**DOI:** 10.1128/mra.00856-23

**Published:** 2023-12-08

**Authors:** Yan Feng, Yu Feng, Zhiyong Zong

**Affiliations:** 1 Center of Infectious Diseases, West China Hospital, Sichuan University, Chengdu, China; 2 Center for Pathogen Research, West China Hospital, Sichuan University, Chengdu, China; 3 Division of Infectious Diseases, State Key Laboratory of Biotherapy, Chengdu, China; Loyola University Chicago, Chicago, Illinois, USA

**Keywords:** pathogen, *Atlantibacter*, *Atlantibacter hermannii*

## Abstract

*Atlantibacter hermannii* is a species of the family *Enterobacteriaceae* and a rare opportunistic pathogen. The draft genome sequence of an *Atlantibacter hermannii* clinical strain that had been isolated from infected muscle tissue was obtained. The genome contains about 4.4 million bases and has no known plasmid replicons.

## ANNOUNCEMENT

*Atlantibacter* is a bacterial genus within the family *Enterobacteriaceae. Escherichia hermannii* has been transferred to this genus as *Atlantibacter hermannii* (1). *A. hermannii* has been implicated as a human pathogen in several cases (2 – 4). Here, we describe the genome sequence of an *A. hermannii* clinical strain. This study was approved by the ethics committee of our hospital with informed consent being waived.

Strain 120144 was recovered from infected muscle tissue, which was sheared, vortexed, and streaked onto blood agar, of a patient with trauma-caused fractures at our hospital in 2019. Genomic DNA was extracted using the QIAamp DNA Blood Mini Kit (Qiagen, Hilden, Germany). Libraries were prepared using the NEBNext Ultra II kit (New England Biolabs, Ipswich, MA) (5). Sequencing (150 bp paired-end reads) was performed on the HiSeq X10 platform (Illumina, San Diego, CA). Raw reads (*n* = 4,691,415 pairs, sequence depth, 320×) were trimmed 10 bp from both ends with Cutadapt v4.0 (6). Adapter removal and quality filtering were performed using bbduk (BBMap v39.01, https://jgi.doe.gov/data-and-tools/software-tools/bbtools, minimum quality, Q15; minimum length, 50 bp). Among the resulted 4,448,349 read pairs (average length, 150 bp), 2,535,455 (depth, 150×) were randomly subsampled using Seqkit v2.5.1 (7) and were assembled using SPAdes v3.15.3 (8).

NCBI Prokaryotic Genome Annotation Pipeline v6.4 (9) was used for annotation. Precise species identification was established based on the average nucleotide identity (ANI) between strain 120144 and type strains of *Atlantibacter* species with JSpeciesWS (10). Plasmid replicons were identified from genome sequences using abricate v1.0.1 (https://github.com/tseemann/abricate) to query PlasmidFinder (11). Its draft genome comprises 4,402,236 bp (34 contigs; N*50*, 430,342 bp; GC%, 54.15%) and has no known plasmid replicons. Strain 120144 belongs to *A. hermannii*, sharing 98.79% ANI with *A. hermannii* DSM 4560^T^ (accession number CP065700). Strain 120144 has *bla*_HERA-1_ [a narrow-spectrum β-lactamase-encoding gene intrinsic to *A. hermannii* (12)] and no other known antimicrobial resistance genes. Strain 120144 was resistant to ampicillin, nitrofurantoin, piperacillin, and ticarcillin and was susceptible to amikacin, amoxicillin/clavulanate, ampicillin/sulbactam, aztreonam, cefepime, cefotaxime, cefpodoxime, ceftazidime, ceftriaxone, cefuroxime, ciprofloxacin, co-trimoxazole, doripenem, ertapenem, gentamicin, imipenem, levofloxacin, meropenem, minocycline, moxifloxacin, piperacillin/tazobactam, tetracycline, ticarcillin/clavulanate, tigecycline and tobramycin as determined using Vitek II (bioMérieux, Marcy-l'Étoile, France).

**Fig 1:**
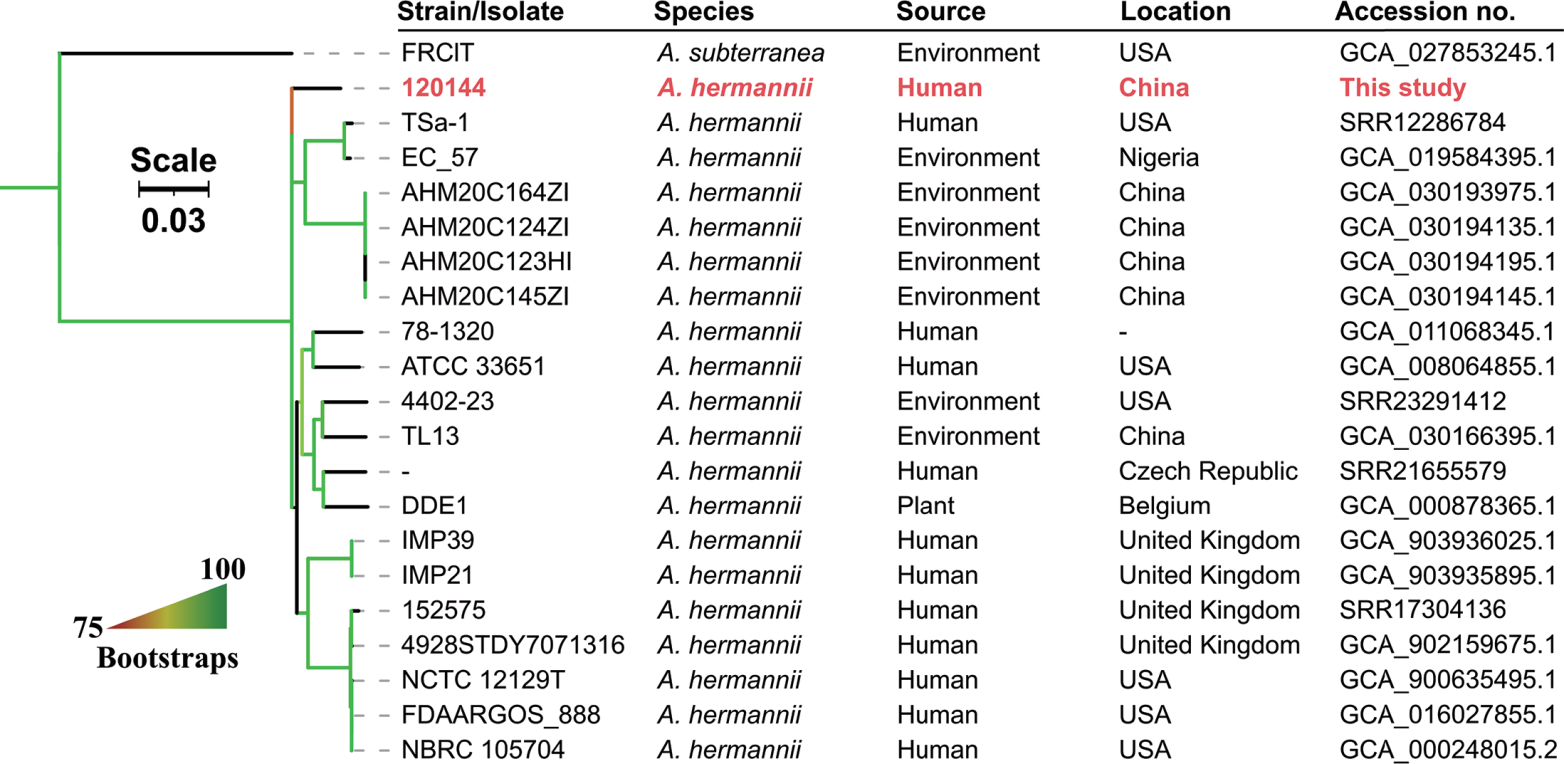
Phylogenomic tree of *A. hermannii.* Strain 120144 is labeled in red. Phylogeny was inferred from concatenated SNP alignments using IQ-Tree v2.2.3 under GTR model applied with gamma distribution and 1,000 bootstrap tests.

To study the clonal relatedness of strain 120144 with other *A. hermannii* strains, we retrieved all *A. hermannii* genome assemblies (*n* = 15) and SRA reads (*n* = 9) from NCBI until 01-09-2023. SRA reads were assembled as described above. Duplicated genomes (*n* = 5) were removed. *A. hermannii* genomes were mapped against the complete chromosome of strain TL13 (accession no. CP126339) using Snippy v4.6.0 (https://github.com/tseemann/snippy) to obtain pairwise distance. Phylogeny was inferred from concatenated single-nucleotide polymorphism (SNP) alignment using IQ-Tree v2.2.3 (13) under GTR model applied with gamma distribution and 1,000 bootstrap tests. Strain 120144 was most closely related to strain EC_57 (accession no. GCA_019584395) (Fig. 1) with 33,468 SNPs. This indicates that strain 120144 is phylogenetic divergent from all other *A. hermannii* strains with available genome sequences in GenBank. Notably, only a few *A. hermannii* genomes are available*,* and more sequencing data are required to study this opportunistic pathogen.

## Data Availability

The whole-genome sequence of 120144 has been deposited in GenBank under the accession no. JAVHXB010000000 and BioSample accession no. SAMN37176092. The Illumina sequence reads have been deposited in the Sequence Read Archive (SRA) database under accession no. SRR25773560.

## References

[1] Hata H , Natori T , Mizuno T , Kanazawa I , Eldesouky I , Hayashi M , Miyata M , Fukunaga H , Ohji S , Hosoyama A , Aono E , Yamazoe A , Tsuchikane K , Fujita N , Ezaki T . 2016. Phylogenetics of family Enterobacteriaceae and proposal to reclassify Escherichia hermannii and Salmonella subterranea as Atlantibacter hermannii and Atlantibacter subterranea gen. nov., comb. nov. Microbiol Immunol 60:303–311. doi:10.1111/1348-0421.12374 26970508

[2] Sedlock C , Tokarczyk M , Sternlieb M , Flomenberg P . 2018. PICC-associated infection with Escherichia hermannii: a case report and review of the literature. IDCases 13:4. doi:10.1016/j.idcr.2018.e00444 PMC6117949 30181953

[3] Choudhury S , Seet C . 2013. Escherichia hermannii bloodstream infection in a long-term haemodialysis patient. Pathology 45:531. doi:10.1097/PAT.0b013e3283633fd7 23856851

[4] Compton J , Wynn M , Willey MC , Sekar P . 2019. Escherichia hermannii as the sole cause of osteomyelitis in a patient with an open tibial shaft fracture. BMJ Case Rep 12:e231206. doi:10.1136/bcr-2019-231206 PMC6887438 31776149

[5] Wang C , Feng Y , Zong Z . 2023. Complete genome sequence of an mcr-10-carrying Enterobacter roggenkampii strain isolated from a human blood culture. Microbiol Resour Announc 12:e0007523. doi:10.1128/mra.00075-23 36912631 PMC10112211

[6] Martin M . 2011. Cutadapt removes adapter sequences from high-throughput sequencing reads. EMBnet J 17:10. doi:10.14806/ej.17.1.200

[7] Shen W , Le S , Li Y , Hu F . 2016. Seqkit: a cross-platform and ultrafast toolkit for FASTA/Q file manipulation. PLoS One 11:e0163962. doi:10.1371/journal.pone.0163962 27706213 PMC5051824

[8] Prjibelski A , Antipov D , Meleshko D , Lapidus A , Korobeynikov A . 2020. Using SPAdes de novo assembler. Curr Protoc Bioinformatics 70:e102. doi:10.1002/cpbi.102 32559359

[9] Tatusova T , DiCuccio M , Badretdin A , Chetvernin V , Nawrocki EP , Zaslavsky L , Lomsadze A , Pruitt KD , Borodovsky M , Ostell J . 2016. NCBI prokaryotic genome annotation pipeline. Nucleic Acids Res 44:6614–6624. doi:10.1093/nar/gkw569 27342282 PMC5001611

[10] Richter M , Rosselló-Móra R , Oliver Glöckner F , Peplies J . 2016. JspeciesWS : a web server for prokaryotic species circumscription based on pairwise genome comparison. Bioinform 32:929–931. doi:10.1093/bioinformatics/btv681 PMC5939971 26576653

[11] Carattoli A , Zankari E , García-Fernández A , Voldby Larsen M , Lund O , Villa L , Møller Aarestrup F , Hasman H . 2014. In silico detection and typing of plasmids using plasmidfinder and plasmid multilocus sequence typing. Antimicrob Agents Chemother 58:3895–3903. doi:10.1128/AAC.02412-14 24777092 PMC4068535

[12] Beauchef-Havard A , Arlet G , Gautier V , Labia R , Grimont P , Philippon A . 2003. Molecular and biochemical characterization of a novel class A beta-lactamase (HER-1) from Escherichia hermannii. Antimicrob Agents Chemother 47:2669–2673. doi:10.1128/AAC.47.8.2669-2673.2003 12878539 PMC166072

[13] Minh BQ , Schmidt HA , Chernomor O , Schrempf D , Woodhams MD , von Haeseler A , Lanfear R . 2020. IQ-TREE 2: new models and efficient methods for phylogenetic inference in the genomic era. Mol Biol Evol 37:2461. doi:10.1093/molbev/msaa131 32011700 PMC7182206

